# Complete Colonic Duplication and Perineal Fistula: Transanal Mucosectomy of the Ectopic Rectum

**DOI:** 10.1055/s-0042-1750028

**Published:** 2022-08-16

**Authors:** Johannes W. Duess, Peter Zimmermann, Franz W. Hirsch, Daniel Graefe, Martin Lacher, Jan-Hendrik Gosemann

**Affiliations:** 1 Department of Pediatric Surgery, University of Leipzig, Leipzig, Germany; 2 Department of Pediatric Radiology, University of Leipzig, Leipzig, Germany

**Keywords:** colonic duplication, mucosectomy, continence

## Abstract

**Background** Colonic duplication may present in different anatomic variants. The surgical approach towards these anomalies can be challenging and has implications for subsequent future continence.

**Case Description** We report on a 1-year-old girl with congenital heart defect and pacemaker who was referred to us with an anorectal malformation. The patient was stooling from both an anus and a perineal fistula. Examination under anesthesia revealed an orthotopic and age-appropriate sized anus with surrounding sphincter and a second rectal lumen ending as a perineal fistula. A computed tomography and contrast enema indicated colonic duplication. Exploratory laparotomy showed a duplicated terminal ileum leading to two ceca and appendices, which joined to a duplicated colon with a septum and common mesentery. At the rectosigmoid junction, one part of the duplication ended as a perineal fistula, the second one led to the (orthotope) anus. The common colonic wall was divided using a stapler. The rectal duplication leading to the perineal fistula was not completely resected but treated by mucosectomy only (Soave plane) leaving its muscular cuff in place. Finally, an ileostomy was created. The postoperative course was uneventful. A contrast enema prior to ostomy takedown demonstrated a well-configurated colon and rectum without stenosis or impaction. The girl is currently continent with a complete resolution of her constipation.

**Conclusion** In cases of complete colonic duplication division of the common wall is simple and safe. Mucosectomy of the ectopic rectum limits pelvic dissection and preserves the entire muscular wall of the duplicated orthotope rectum.

## Introduction

Duplications of the alimentary tract are rare congenital malformations that can occur in any part of the gastrointestinal tract. Duplications mostly affect the esophagus and ileum with only 4 to 18% involving the colon. 1 They typically share a common wall and vascular blood supply with the intestine. The clinical presentation varies according to the location and extent of the lesion, as well as the type of mucosal lining. The majority of enteric duplications is cystic (75%) with no communication to the adjacent alimentary tract while the remaining is tubular, and may communicate with the intestinal lumen. 2 The treatment of choice is complete resection of the duplication. In tubular duplication of the colon a total or subtotal colectomy is often performed. 3 The surgical approach towards these anomalies can be challenging and has implications for subsequent future continence. 1 2 3 4 We report the case of a girl with total colonic duplication and a perineal fistula.

## Case Report

A 1-year-old girl with congenital heart defect and pacemaker was referred to our institution with severe constipation and a suspected anorectal malformation.

The child was born via cesarian section at 36 1/7 gestational age (birth weight 2,430 g) at an outside hospital. Postnatal examination revealed an orthotopic anus with a perineal fistula and passage of meconium via both orifices. The sacrum was normal to palpation. Ultrasound excluded tethered cord and a renal pathology. Due to low oxygen saturations between 90 and 95%, an echocardiography revealed complete atrioventricular septal defect. Chromosome analysis was normal.

At the age of 4 months, the girl underwent cardiac surgery followed by insertion of a pacemaker 1 month later. On examination under anesthesia after 3 months both the orthotopic rectum and the perineal fistula could be intubated with Hegar dilatators without connection of the two orifices.

**Fig. 1:**
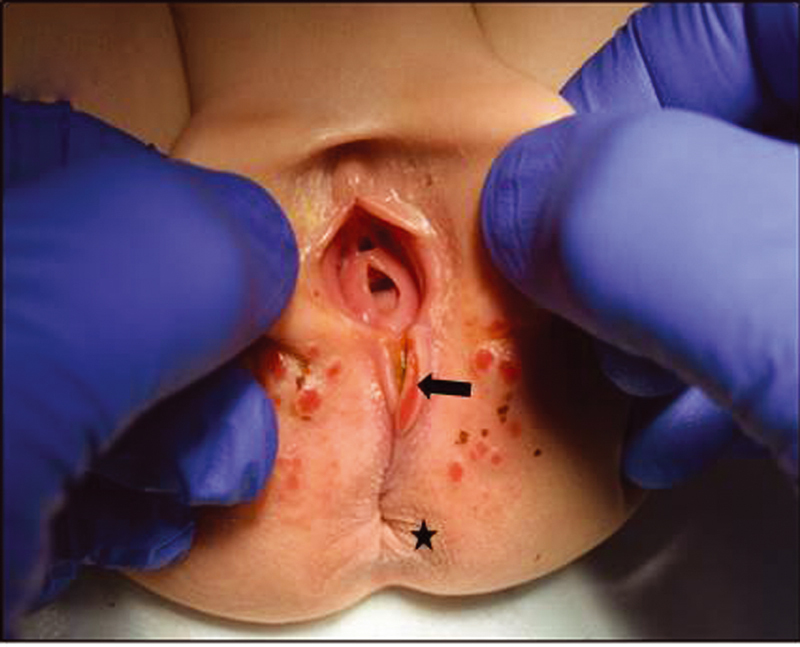
Orthotopic and age-appropriate sized anus with surrounding sphincter (asterisk) and a perineal fistula (arrow).

**Fig. 2:**
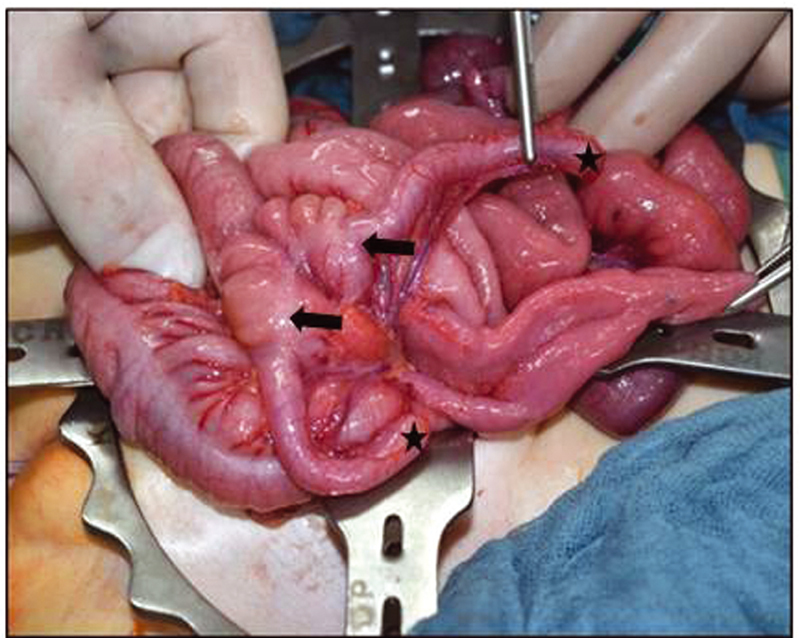
Duplicated terminal ileum with a separated mesentery leading to two ceca (arrows) and appendices (asterisks).

**Fig. 3:**
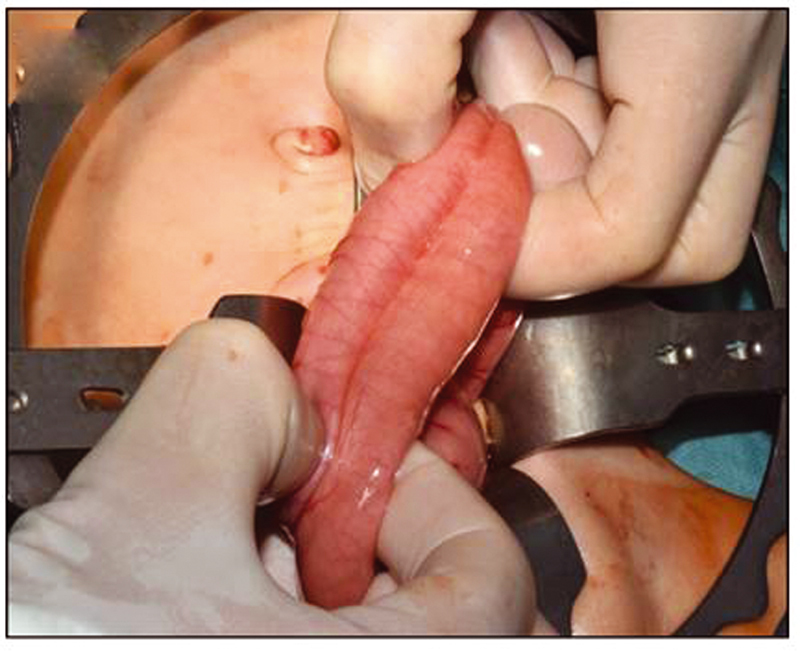
Completely duplicated colon with a septum and common mesentery.

**Fig. 4:**
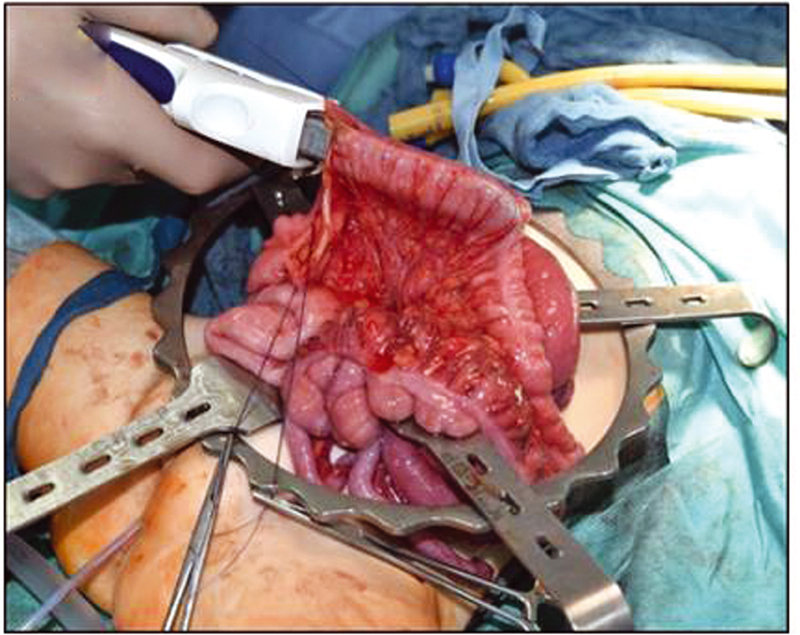
Division of the common colonic wall using a stapler to create one lumen up to the level of the ileocaecal valve.

**Fig. 5:**
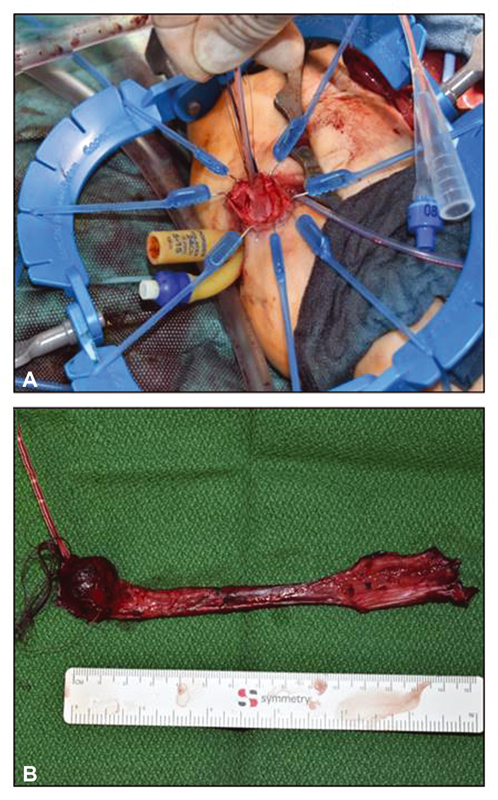
( **A** and B) Mucosectomy (Soave plane) of the rectal duplication leading to the perineal fistula leaving its muscular cuff in place.

**Fig. 6:**
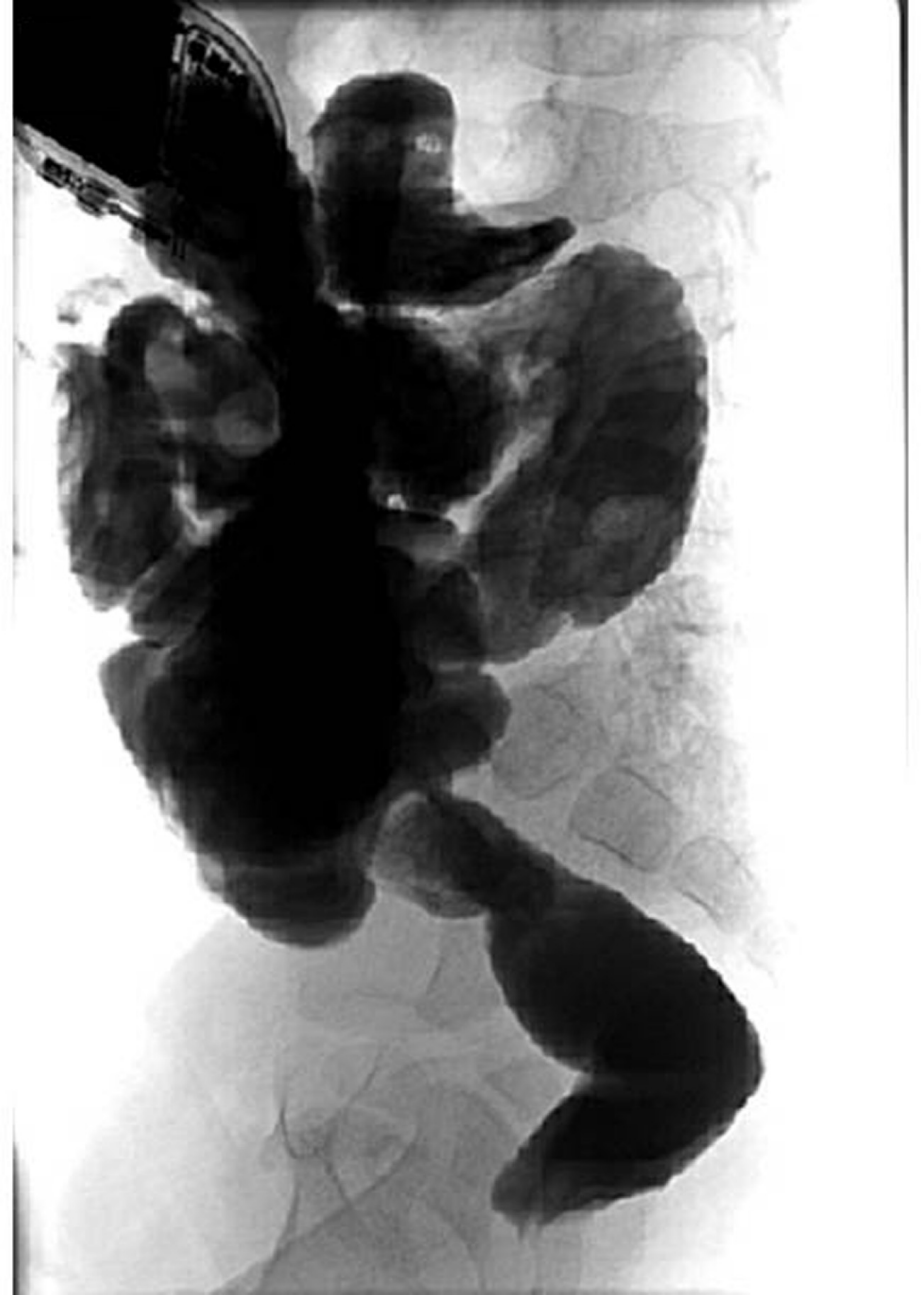
Contrast enema 6 weeks postoperatively demonstrating a well-configured colon and rectum without stenosis or impaction.

The child was referred to our department at the age of 13 months because of the congenital anomaly with severe constipation. Examination under anesthesia and electrostimulation confirmed an orthotopic and age-appropriate sized anus with surrounding sphincter and a perineal fistula ( Fig. 1 ). The fistula could be intubated with a Hegar dilatator size 6 and the anus with a size 12, suggesting a rectal duplication. Massive coprostasis was also noted, especially in the perineal fistula. The child was started on rectal irrigation. A computed tomography (CT) and contrast enema indicated a colonic duplication up to the splenic flexure. Exploratory laparotomy revealed a duplicated terminal ileum starting 10 cm proximal to the ileocecal valve with a separated mesentery leading to two ceca and appendices ( Fig. 2 ), which joined to a completely duplicated colon with a septum and common mesentery ( Fig. 3 ). The rectosigmoid was completely divided. One part of the duplication ended as a perineal fistula, the second one led to the orthotopic anus. The common colonic wall was divided using a stapler to create one lumen up to the level of the ileocaecal valve ( Fig. 4 ). The ceca were left in place. Multiple colostomies were required to allow passage of the stapling device. The rectal duplication which led to the perineal fistula was closed by a simple mucosectomy (Soave plane) from proximal to distal leaving its muscular cuff in place ( Fig. 5A , B ). Finally, a protective ileostomy was created. The postoperative course was uneventful and the girl was discharged after 13 days following surgery. A contrast enema 6 weeks postoperatively demonstrated a well-configured colon and rectum without stenosis or impaction ( Fig. 6 ). Ileostomy takedown was performed 1 month later. After 20 months of follow-up the girl is doing well with regular and normal bowel movements.

## Discussion

Congenital duplications of the alimentary tract are rare anomalies with a reported incidence of approximately 1 in 4,500 live births. 2 5 6 The embryological origin of these lesions still remains unknown and several theories have been proposed: One theory describes undetermined environmental factors such as trauma and hypoxia with adverse effects in early fetal development, which are often associated with malrotation and atresia. According to another one the hindgut anlage splits leading to a duplication of the terminal portion of the gastrointestinal tract. 1 7

Duplications of the large bowel constitute approximately 13% of all duplications and are divided into two subgroups, including cystic and tubular duplications. The duplicated segment may be present on the mesenteric or antimesenteric side of the colon, sharing vascular blood supply and a common wall with the intestine, although each has its own mucosal lining. Symptoms and clinical presentation may depend on the location and size of the lesion, including intestinal bleeding, abdominal pain, and vomiting, or it may be an incidental finding on physical examination. 1 2 7 8 In rare cases, colorectal tubular duplications are associated with anorectal malformations, duplication of internal or external genitalia, as well as vertebral anomalies. Therefore, the operative management can be complex and has implications for future continence. 2 9

The patient in our case report was initially suspected to have an anorectal malformation and presented with an orthotopic anus and perineal fistula passing meconium from both orifices. However, when an examination under anesthesia was performed the fistula could be intubated with a Hegar dilator size 6. To define the preoperative anatomy a CT, which was chosen due to the cardiac pacemaker, and contrast enema were conducted showing colonic duplication.

Alimentary tract duplications may represent a diagnostic challenge as their clinical and anatomical presentation can be variable requiring selective radiologic imaging studies to determine the precise anatomy. 9 10 Diagnostics often rely on ultrasonography, barium swallow, CT, or magnetic resonance (MR) imaging scans. 2 Furthermore, these studies may also demonstrate associated anomalies. 11 MR scan is the preferable method in the pediatric population and can identify any spinal abnormalities, whereas a barium enema may accurately delineate the anatomy of the duplicated colon. 11 12 Therefore, a thorough preoperative planning is mandatory.

The present combination of an anorectal malformation with complete duplication of the colon and rectum is uncommon with only few case series or reports being available in the literature. Jellali et al presented a case series of three patients with anorectal malformation and complete tubular colonic duplication. In all patients the anorectal malformation was discovered at birth, whereas the colonic duplication was found during creation of colostomy preceding the pull-through procedure. 13 AbouZeid et al have recently published a case of a 2-year-old girl who was found to have a variant of anorectal anomaly associated with complete colonic duplication. The duplicated colon was also initially missed and the patient was treated as an anorectal malformation with perineal fistula by posterior-sagittal anorectoplasty. However, the child presented later again with abnormal passage of stools through her vestibule and a duplicated colon with perineal and vestibular fistula was subsequently diagnosed. 14 Fistulae are reported to occur in up to 20% of cystic rectal duplications. 15 Furthermore, Yousefzadeh et al described five entities of tubular colonic duplications, using the existence of a fistula as the major discriminator: group 1 as duplication with two perineal ani, group 2 and 3 as duplication with fistula(e) in females and males, respectively, group 4 as duplication with imperforate anus and without fistula, and group 5 as communicating duplication with single perineal anus. 9 16 17

Several surgical approaches have been described in the management of long segment colonic duplication with shared blood supply. We performed an exploratory laparotomy which revealed a long segment of duplicated colon including the terminal ileum with two ceca and appendices, which joined to a completely duplicated colon with one septum and common mesentery. The common colonic wall was simply divided using a stapling device as successfully described in previous case reports. 1 3 14 18 The duplicated ceca and appendices were left in place. In long segment colonic duplication, fenestrating the two lumens by hand-sewn side-to-side anastomosis or linear stapler as performed in our case is recommended in the majority of patients. 1 3 4 19 A large communication between the duplicated and normal large bowel may also ensure emptying of stool as patients do also present with compressive symptoms such as constipation. 3 8 In contrast to long segment types which share a common blood supply with the normal intestine duplications limited to a short segment or cystic duplications should be managed by complete resection. 1 2

Division of the common wall of the duplicated rectum and resection of the fistula can be challenging and may require an extensive pelvic dissection potentially violating healthy tissue. On the other hand, leaving a small blind pouch of rectum may result in fecal impaction, constipation, or a recurrent fistula. 20 In our patient, the rectal duplication leading to the perineal fistula was not resected but treated by mucosectomy (Soave plane) leaving its muscular cuff in place. Limited surgical dissection may prevent compromise of the pelvic floor. Mucosectomy has been shown to be successful for the treatment of rectal duplications. 4 9 11 14 20 A similar approach has been described by AbouZeid et al in the patient with anorectal anomaly and complete colonic duplication. Following creation of a single wide lumen using a linear cutting stapler for the common wall of the duplicated colon a mucosectomy was performed in the distal colon and rectum communicating with the vagina, followed by seromuscular closure. 14

The majority of case reports have shown excellent functional outcomes in terms of regular bowel movements and also satisfying cosmetic results following surgery for colonic duplication. Only few authors have described postoperative complications, including reoperation for incomplete division of the distal rectal septum and death due to ongoing sepsis. 1 21 Our patient had a contrast enema prior to reversal of the ileostomy demonstrating a well-configured colon and rectum without stenosis or impaction. At the age of 3 years (20 months after surgery) the girl is doing well and has no signs of constipation.

## Conclusion

We report on a 1-year-old girl with a very rare association of complete colorectal duplication with perineal fistula. Preoperative diagnosis was made by examination under anesthesia, CT, and contrast enema. For the management of colonic duplication, a single lumen was created by simple division of the common wall, followed by mucosectomy of the distal ectopic rectum.

The association of anorectal malformation with complete colonic duplication is rare. Therefore, careful preoperative examination is vital to avoid unexpected findings during surgery. Limited dissection in the submucosal plane preserves the muscular wall of the duplicated orthotopic rectum to ensure normal future continence.

## Notes

New Insights and Importance for a Pediatric Surgeon Mucosectomy of the ectopic rectum limits pelvic dissection and preserves the entire muscular wall of the duplicated orthotope rectum.
